# Epitaxial Core/Shell Nanocrystals of (Europium-Doped) Zirconia and Hafnia

**DOI:** 10.1021/jacs.4c05037

**Published:** 2024-07-22

**Authors:** Carlotta Seno, Nico Reichholf, Francesco Salutari, Maria Chiara Spadaro, Yurii P. Ivanov, Giorgio Divitini, Alexander Gogos, Inge K. Herrmann, Jordi Arbiol, Philippe F. Smet, Jonathan De Roo

**Affiliations:** † Department of Chemistry, University of Basel, Mattenstrasse 24a, 4058 Basel, Switzerland; ‡ Catalan Institute of Nanoscience and Nanotechnology (ICN2), CSIC and BIST, 08193 Barcelona, Catalonia, Spain; § Department of Physics and Astronomy “Ettore Majorana”, University of Catania and CNR-IMM, Via S. Sofia 64, 95123 Catania, Italy; ∥ Electron Spectroscopy and Nanoscopy, Istituto Italiano di Tecnologia, Via Morego 30, 16163 Genova, Italy; ⊥ ICREA, 08010 Barcelona, Catalonia, Spain; # Laboratory for Particles-Biology Interactions, Department of Materials Meet Life, Swiss Federal Laboratories for Materials Science and Technology (Empa), Lerchenfeldstrasse 5, 9014 St. Gallen, Switzerland; ∇ Nanoparticle Systems Engineering Laboratory, Institute of Process Engineering, Department of Mechanical and Process Engineering, ETH Zurich, Sonneggstrasse 3, 8092 Zurich, Switzerland; ○ LumiLab, Department of Solid State Sciences, Ghent University, Krijgslaan 281-S1, 9000 Ghent, Belgium

## Abstract

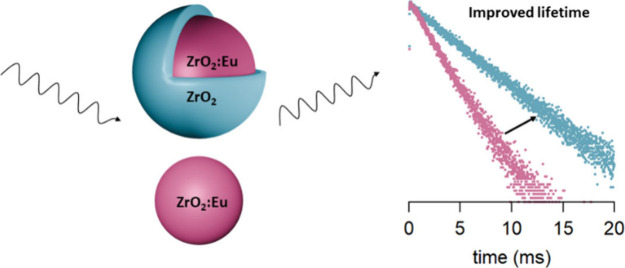

A careful design of the nanocrystal architecture can strongly enhance the nanocrystal function. So far, this strategy has faced a synthetic bottleneck in the case of refractory oxides. Here we demonstrate the epitaxial growth of hafnia shells onto zirconia cores and pure zirconia shells onto europium-doped zirconia cores. The core/shell structures are fully crystalline. Upon shelling, the optical properties of the europium dopant are dramatically improved (featuring a more uniform coordination and a longer photoluminescence lifetime), indicating the suppression of nonradiative pathways. These results launch the stable zirconium and hafnium oxide hosts as alternatives for the established NaYF_4_ systems.

Zirconium and hafnium oxide nanocrystals (NCs) are appealing (host) materials due to their high thermal and chemical stability and large band gap.^1^ Colloidal ZrO_2_ NCs are components for (in)organic composites,^2−4^ while HfO_2_ NCs find applications in memory devices.^5,6^ Due to the high atomic number of hafnium and the high density of hafnia, HfO_2_ NCs have been developed as computed tomography contrast agents,^7−9^ scintillators,^10^ and radiation therapy enhancers.^11−13^

Both ZrO_2_ and HfO_2_ NCs serve as hosts for optically active lanthanide ions, e.g., europium.^1,14−18^ Fluorides (e.g., NaYF_4_ and NaGdF_4_) are another class of nanocrystals that are widely used as hosts for lanthanides, with application in upconversion and downconversion.^19−23^ In the fluoride system, the syntheses are well-developed, allowing for the precise positioning of dopants inside the nanocrystal and the growth of undoped shells on the doped core. The latter results in core/shell architectures, which were pioneered in the field of semiconductor nanocrystals (quantum dots) to prevent the excited electrons and holes from interacting with surface traps.^24,25^ Likewise, shelling protects the lanthanides from surface effects and thus increases the quantum efficiency of both up- and downconversion processes.^26^ Additionally, multilayered structures offer controlled energy cascades in the case of lanthanide-doped fluorides.^27^ Higher quantum efficiencies coupled with long lifetimes enabled their use in, e.g., time-gated fluorescence imaging.^15,28^ The oxide hosts have found less widespread use because of the synthetic challenge of producing colloidally stable oxide nanocrystals with a complex (e.g., core/shell) architecture.^29^ However, the oxide hosts are more chemically stable, while the fluorides dissolve in highly dilute aqueous media.^30^

Surfactant-assisted, nonaqueous synthesis methods have allowed the synthesis of ZrO_2_ and HfO_2_ NCs with control over their size.^1,31−37^ Even more, solid solutions of various compositions (Zr_*x*_Hf_1–*x*_O_2_) were obtained as colloidal nanocrystals.^34,38,39^ Hf_0.5_Zr_0.5_O_2_ is an interesting ferroelectric material, making it promising for nonvolatile memory devices.^40,41^

Here we leverage the control of nonaqueous synthesis and report metal oxide core/shell nanocrystals. We epitaxially grow HfO_2_ (or ZrO_2_) onto ZrO_2_ and ZrO_2_ onto ZrO_2_:Eu^3+^. The beneficial effect of the shell on the optical properties of the europium dopant is established. Having access to these novel heterostructures, which are unavailable in bulk, opens up possibilities for their application in different areas, from microelectronics to scintillators.

Zr and Hf are chemically very similar, and both oxide nanocrystals can be synthesized at 340 °C from the metal chloride and isopropoxide in tri-*n*-octylphosphine oxide (TOPO).^1^ Under these conditions, HfO_2_ forms nanorods with the monoclinic crystal structure, while ZrO_2_ forms spherical nanocrystals with the tetragonal crystal structure. We first focus on the ZrO_2_/HfO_2_ core/shell structure, since one can conveniently distinguish growth of HfO_2_ onto the spherical ZrO_2_ cores from separately nucleating HfO_2_ nanorods. ZrO_2_ and HfO_2_ form similar crystal structures (monoclinic, tetragonal, and cubic) with negligible lattice mismatch, thus allowing for epitaxial growth. We followed a two-step approach toward the core/shell structure. In the first step, we synthesized and isolated ZrO_2_ cores from ZrCl_4_ and Zr(O^i^Pr)_4_·^i^PrOH in TOPO (Figure 1A).^42^ The cores are spherical with an average diameter of 3.8 nm according to bright-field transmission electron microscopy (BF TEM) (Figure 1B). The cores have the tetragonal (*P*4_2_/*nmc*) crystal structure (Figures S1 and S2), and their surface is covered with a mixture of protonated TOPO, dioctyl phosphinate, and dioctyl pyrophosphonate (Figure 1D).^42^ In a second step, the purified cores were added to a new reaction mixture of HfCl_4_(THF)_2_ and Hf(O^i^Pr)_4_·^i^PrOH in TOPO (see Figure 1A). After 2 h at 340 °C, the core/shell structures were isolated and purified. The nanocrystal surface is covered by the same ligands as mentioned before (see Figure 1D for the ^31^P NMR spectrum). Atomic-resolution high-angle annular dark-field (HAADF) scanning TEM (STEM) analysis shows that the resulting nanocrystals are no longer spherical but more clearly faceted, exposing {011̅} and {101̅} surfaces (Figures S3 and S4). To be able to compare the sizes of core and core/shell particles, we analyze the BF TEM image (Figure 1C) by measuring the projected area of the faceted nanocrystals and calculating an apparent “diameter”, which is the diameter for a circle with equal area. The core/shell structures have an average “diameter” of 4.9 nm, clearly indicating that the cores (*d* = 3.8 nm) have grown to larger sizes. Crystal growth is further confirmed by the narrower reflections in X-ray diffraction (XRD) (Figure S1) and extended features in the pair distribution function (PDF) (Figure S2).

**Figure 1:**
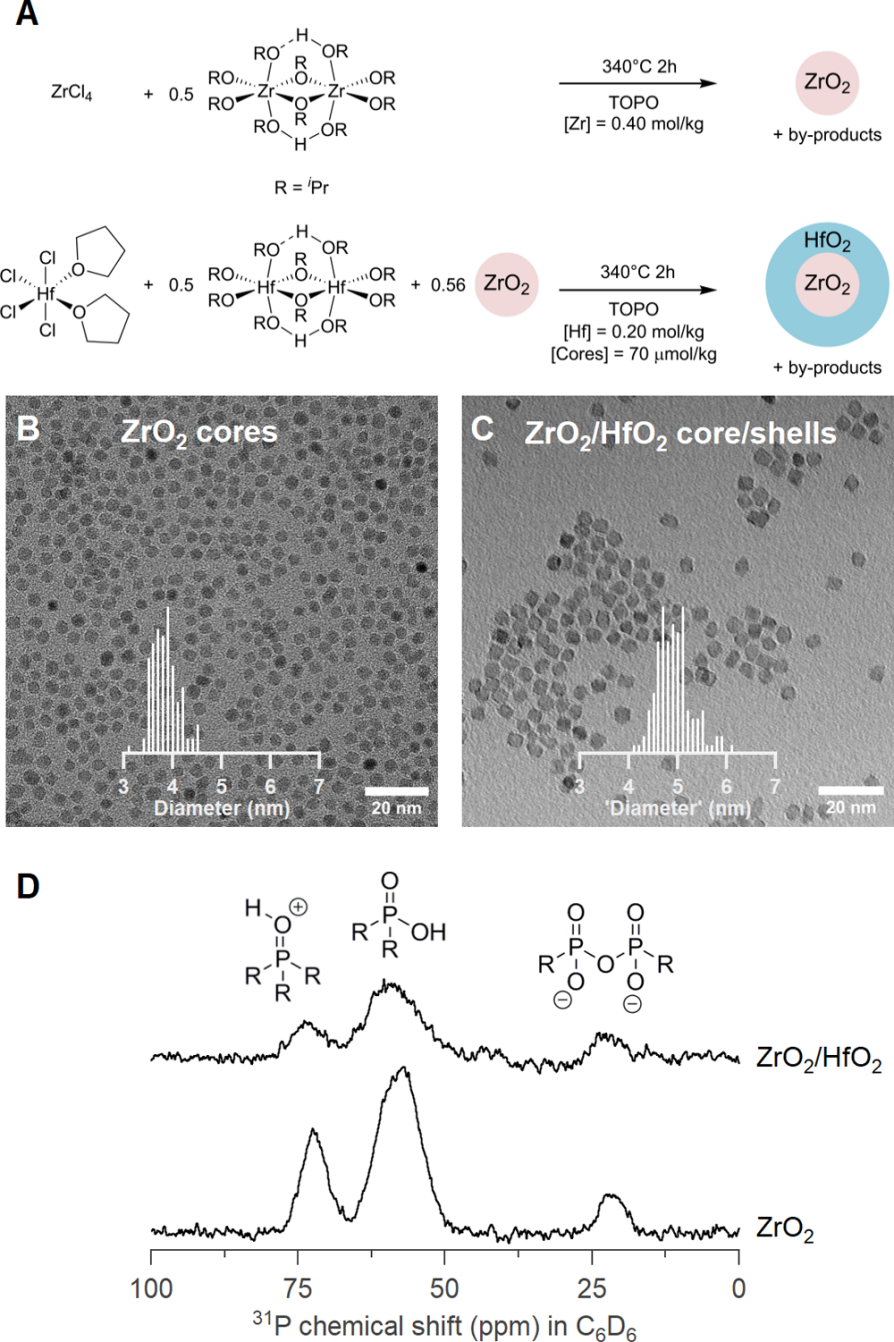
(A) ZrO_2_ core and ZrO_2_/HfO_2_ core/shell synthesis procedure, where the metal chloride, tetrahydrofuran, propene, and isopropanol are obtained as byproducts.^31,32^ (B, C) BF TEM images of (B) ZrO_2_ cores and (C) ZrO_2_/HfO_2_ core/shells. The histograms are based on more than 100 particles. (D) Solution ^31^P NMR spectra of ZrO_2_ and ZrO_2_/HfO_2_ NCs.

The core/shell interface was further analyzed by atomic-resolution HAADF STEM (see Figures 2, S3, and S4). From the contrast in Figure 2A, we can recognize the presence of a core/shell structure, as the shell appears brighter with respect to the core due to the higher atomic number of Hf compared to Zr. The power spectrum (FFT) analysis in the reciprocal space shows that the ZrO_2_ core has the *P*4_2_/*nmc* tetragonal structure and that the HfO_2_ shell has a similar structure. Tetragonal HfO_2_ has the following lattice parameters at room temperature: *a* = *b* = 3.65 Å, *c* = 5.33 Å. These are slightly higher than those of tetragonal ZrO_2_: *a* = *b* = 3.59 Å, *c* = 5.18 Å.^43,44^ This is in good agreement with the observed crystal arrangement in our core/shell nanoparticles. The (101) and (101̅) reflections are “elongated” or doubled, indicating the presence of HfO_2_ that grows epitaxially and partially relaxed onto the indicated surfaces of the ZrO_2_ core. The frequency-filtered map clearly displays the core in green and the shell in red.

**Figure 2:**
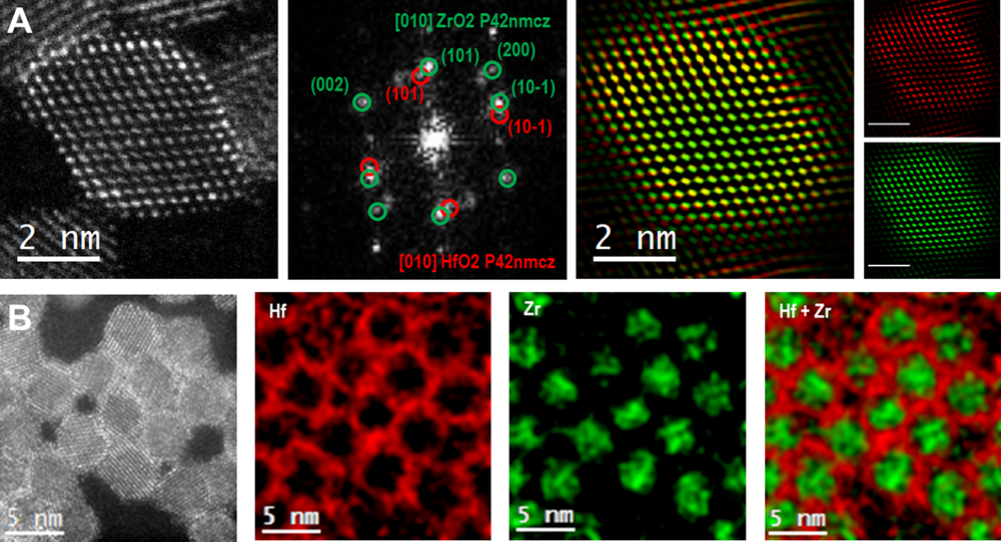
(A) HAADF STEM image of a core/shell ZrO_2_/HfO_2_ nanocrystal, the corresponding power spectrum (FFT) analysis in the reciprocal space along the [010] zone axis, and the frequency-filtered map HAADF STEM image. (B) HAADF STEM image of several ZrO_2_/HfO_2_ nanocrystals and the corresponding EDX compositional maps featuring hafnium and zirconium.

While pure HfO_2_ nanocrystals would crystallize in the monoclinic *P*2_1_/*c* structure, epitaxial growth thus stabilizes hafnia in its tetragonal structure. Final confirmation of the core/shell structure is provided by energy-dispersive X-ray spectroscopy (EDX) compositional mapping, which shows regions with zirconium in the center and hafnium in the shell (Figure 2B). When shelling the zirconia cores with zirconia shells under identical conditions, we cannot infer the success from compositional mapping. However, the final core/shell particles had an average “diameter” of 5.5 nm, indicating successful growth (Figure S5).

Second, we turn to the optically active ZrO_2_:Eu/ZrO_2_ core/shell system. We synthesized europium-doped zirconia (10.0% nominal doping) from europium acetate, zirconium propoxide, and benzyl alcohol (Figure 3A).^15^ After synthesis, we functionalized the nanocrystal surface with dodecanoic acid ligands (Figure S6). The ZrO_2_:Eu cores have an average diameter of 3.5 nm (Figure 3B), possess the tetragonal crystal structure (Figures S1 and S2), and have an actual doping concentration of 9.0% (determined by ICP-OES). The efficient incorporation of europium into the oxide lattice is in agreement with previous reports.^15−17^

**Figure 3:**
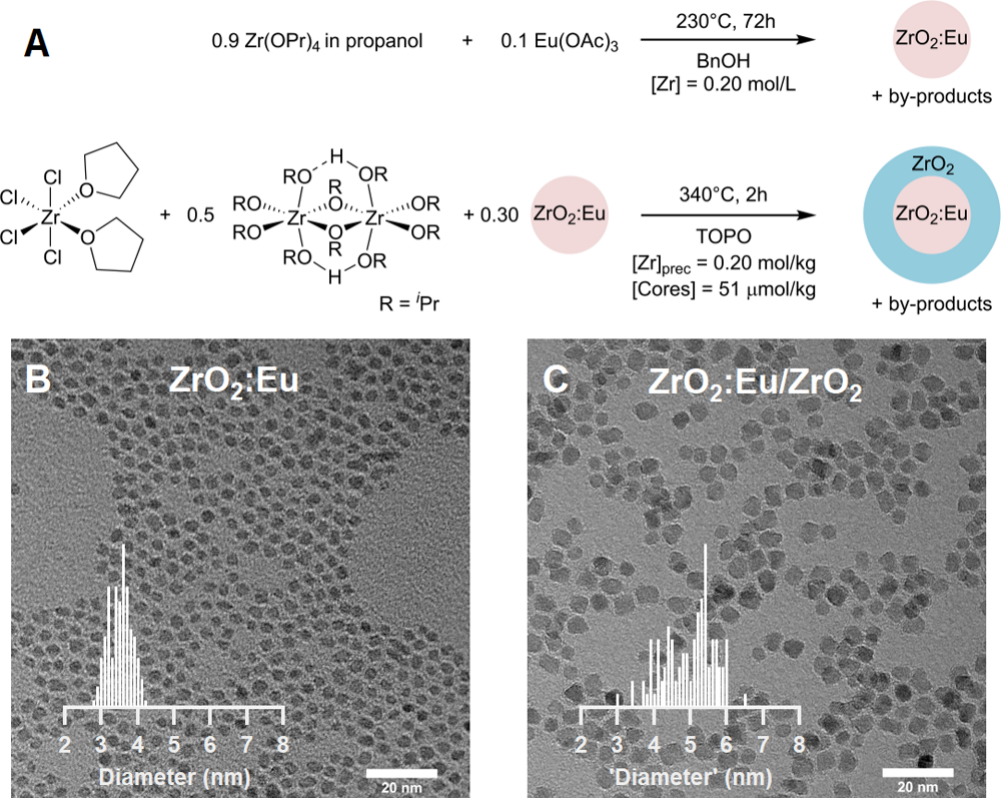
(A) ZrO_2_:Eu core and ZrO_2_:Eu/ZrO_2_ core/shell syntheses. (B, C) BF TEM images of (B) ZrO_2_:Eu cores and (C) ZrO_2_:Eu/ZrO_2_ core/shells. The histograms are based on more than 100 particles.

The cores were subjected to a shelling procedure using ZrCl_4_(THF)_2_ and Zr(O^i^Pr)_4_·^i^PrOH (Figure 3A). After 2 h at 340 °C, colloidally stable nanocrystals were isolated and purified (Figure S7 for ^31^P NMR and Figure S8 for DLS). BF TEM imaging shows an increase in the average “diameter” to 5.1 nm (Figure 3C), and again, sharper reflections in XRD and extended signals in the PDFs are observed, confirming growth of the tetragonal crystal (Figures S1 and S2). HAADF STEM and powder spectrum analysis further confirm that both ZrO_2_:Eu cores and ZrO_2_:Eu/ZrO_2_ core/shells have the tetragonal structure (Figures S9 and S10). The europium content decreases in the core/shells to 1.8% metal content. This is close to the expected 2.1% considering the stoichiometry of the reaction.

The optical properties of the europium dopant change substantially when the cores are shelled (see Figures 4A, S11, and S12). The excitation and emission spectra of Eu in the ZrO_2_:Eu cores is consistent with previous reports of Eu doped into colloidal ZrO_2_ nanocrystals or ZrO_2_ powders.^15,17,45,46^ Upon shelling, certain emission channels disappear (e.g., the emission band at 612 nm, belonging to a ^5^D_0_ → ^7^F_2_ transition), and the remaining emission peaks are more narrow (Figure 4A). This indicates a change in the coordination around the europium ion. This is further demonstrated by the shape of the ^5^D_0_ → ^7^F_0_ transition around 580 nm, measured at 10 K (Figure S13). This *J* = 0 → *J*′= 0 transition is not split by the crystal field, and thus, spectrally different contributions for this transition can be assigned to different coordinating environments. For the core/shell, the emission band corresponding to this transition is narrower and more symmetric, indicating a highly uniform environment for europium.

**Figure 4:**
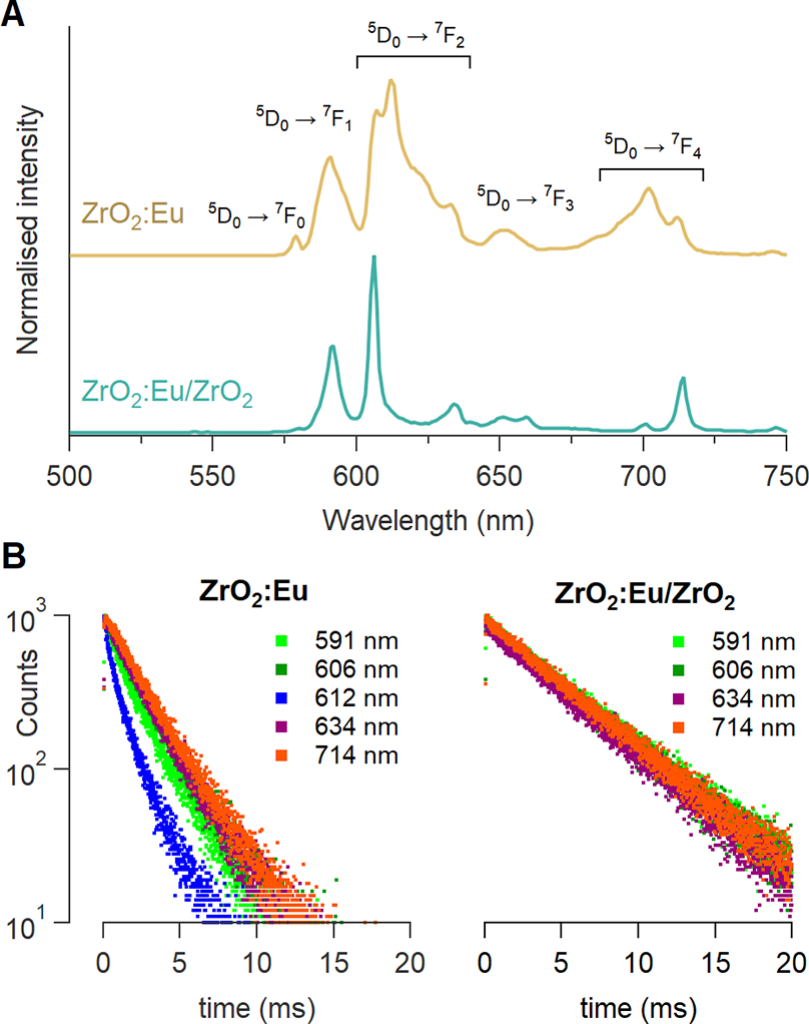
(A) Photoluminescence emission spectra and (B) lifetime decays at various emission wavelengths of ZrO_2_:Eu cores and ZrO_2_:Eu/ZrO_2_ core/shells, excited at 238 nm. The measurements were carried out at room temperature in cyclohexane with an absorbance of 0.1 at 238 nm.

The shell also has a significant impact on the lifetime of the excited state (Figure 4B). For the ZrO_2_:Eu cores, the luminescence decay depends on the monitored emission wavelength but in all cases is biexponential. This variation in lifetime points again to different europium coordination environments. We fit the decay profiles using the function
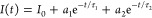
1At 606 nm, we determine a slow component of τ_1_ = 2.7 ± 0.1 ms and a fast component of τ_2_ = 1.2 ± 0.1 ms. The slow component contributes 86% to the total emission, calculated via
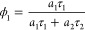
2For the core/shells, the decay is monoexponential and independent of the monitored wavelength (Figure 4B). We determined a lifetime of 5.3 ± 0.1 ms. This is similar to the longest values reported for Eu^3+^ in ZrO_2_ (nano)powders,^47^ indicating that the contribution of nonradiative decay, e.g. via interaction with surface or other defects, is limited. In the literature, long lifetimes of 4–5 ms appear only for low doping percentages (1%). Higher doping percentages are typically associated with shorter lifetimes.^47^ Here we report a core doped with 9% Eu that features a lifetime of 5.3 ms after shelling and thus passivation of the surface defects. Some authors attribute the faster decay to a monoclinic crystal phase and the slower decay to the tetragonal phase.^48^ Given that we find no evidence of a monoclinic phase, we assign the fast decay (present only in the ZrO_2_:Eu cores) to Eu ions that are near the nanocrystal surface. Based on the changes in the emission spectrum and the increase in lifetime, we conclude that the shelling improves the incorporation of the Eu ions in the oxide crystal. Indeed, Eu^3+^ ions act as a probe for their local environment.^49−52^

While excitation of the ZrO_2_:Eu/ZrO_2_ core/shells at 240–260 nm yields only the 4f–4f transitions of Eu^3+^, excitation at 300 nm generates an additional broadband contribution ranging from 350 to 600 nm related to defect emission in ZrO_2_ (Figure S14).^31,53−55^ The coexistence of broadband and europium emission in ZrO_2_ (with 0.4% Eu doping) was recently exploited for ratiometric fluorescence thermometry in the range from 130 to 230 K.^56^ We did not observe this strong broadband emission for the ZrO_2_:Eu cores with high Eu doping (9%) (see Figure S14), implying that the presence of Eu ions suppresses the defect emission. This suggests that for the core/shell particles, the Eu ions remain located in the core during the shelling, since the presence of Eu ions in the shell would suppress the ZrO_2_ defect emission (either by preventing the defect from forming or by short-range energy transfer to Eu).^57−59^ Therefore, the overall emission spectrum of the core/shells contains emission from Eu^3+^ in the core and defect emission from the shells. This also explains the drastic increase in the Eu^3+^ lifetime of the core/shell particles, since the Eu^3+^ ions are now in a perfect environment, shielded from the surface.

In summary, we have reported the use of crystalline zirconium or hafnium oxide to shell nanocrystals. This method was applied to improve the local environment of europium dopants in zirconia nanocrystals and to suppress nonradiative de-excitation pathways. Our results are an invitation to explore intricate nanocrystal architectures with oxide host materials.

## Data Availability

The data that support the findings of this study are openly available in Zenodo at DOI: 10.5281/zenodo.12684072.

## Supporting Information Available

Description of the synthetic procedures and details of general instrumentation; characterization of core and core/shell nanoparticles with HAADF STEM, ^1^H and ^31^P NMR, DLS, powder XRD, PDF, and additional emission and excitation spectra (PDF)
