# Supplementary material for: Epitaxial Core/Shell Nanocrystals of (Europium-Doped) Zirconia and Hafnia
Source: J Am Chem Soc. 2024 Jul 22;146(30):20550–5. doi: 10.1021/jacs.4c05037 (PMC11295171; doi:10.1021/jacs.4c05037)
Supplement: Supplementary file 1 — ja4c05037_si_001.pdf [file ja4c05037_si_001.pdf]

Epitaxial Core/Shell Nanocrystals of (Europium-Doped) Zirconia and Hafnia

Carlotta Seno[§], Nico Reichholf[§], Francesco Salutari[†], Maria Chiara Spadaro[†,≠], Yurii P. Ivanov[¶], Giorgio Divitini[¶], Alexander Gogos[¤,#], Inge K. Herrmann[¤,#], Jordi Arbiol[†,ǂ], Philippe F. Smet[‡] and Jonathan De Roo[§]*

§ Department of Chemistry, University of Basel, Mattenstrasse 24a, 4058 Basel, Switzerland

† Catalan Institute of Nanoscience and Nanotechnology (ICN2), Barcelona 08193 Catalonia, Spain

≠ Department of Physics and Astronomy 'Ettore Majorana', University of Catania and CNR-IMM, Via S. Sofia 64, 95123 Catania, Italy

¶ Electron Spectroscopy and Nanoscopy, Istituto Italiano di Tecnologia, Via Morego 30, 16163 Genova, Italy

¤ Laboratory for Particles-Biology Interactions, Department of Materials Meet Life, Swiss Federal Laboratories for Materials Science and Technology (Empa), Lerchenfeldstrasse 5, 9014 St. Gallen, Switzerland

Nanoparticle Systems Engineering Laboratory, Institute of Process Engineering, Department of Mechanical and Process Engineering, ETH Zurich, Sonneggstrasse 3, 8092 Zurich, Switzerland

ǂ ICREA, Barcelona 08010 Catalonia, Spain

‡ LumiLab, Department of Solid State Sciences, Ghent University, Krijgslaan 281-S1, Ghent, 9000, Belgium

Experimental

Materials

$ZrCl_4$ (99.9%) and $HfCl_4$ (99.9%) were purchased from Strem Chemicals. $ZrCl_4(THF)_2$ and $HfCl_4(THF)_2$ were synthesized following the procedure reported by Manzer *et al.*[1] $Zr(O^iPr)_4$ ^iPrOH and $Hf(O^iPr)_4$ ^iPrOH were synthesized following the procedure reported firstly by Bradley *et al.*[2] and modified by Dhaene *et al.*[3] Zirconium propoxide in 1-propanol (70 wt. %) was purchased by Sigma-Aldrich and $Eu(OAc)_3$ (99.9%) by Strem Chemicals. Tri-*n*-octylphosphine oxide (Strem Chemicals, 99%) was recrystallized according to the procedure described by Owen *et al.*[4] Benzene-d_6 (Apollo Scientific, 99.5%) was used for NMR characterization. Toluene (VWR chemicals, for HPLC 100 %), cyclohexane (Honeywell, for HPLC ≥ 99.7 %), acetone (Biosolve Chemicals), diethyl ether (VWR chemicals, for HPLC ≥ 99.5 %) and dodecanoic acid (Sigma Aldrich, ≥ 98%) were used without further purifications. Benzyl alcohol (Sigma Aldrich, 99.9 %) was dried overnight with magnesium sulfate (Acros Organics, 99%), filtered and vacuum distilled at the Schlenk line into a Strauss flask before being loaded into the glovebox for storage. PTFE membranes (Acrodisc PSF witth GXF/0.2 µm Supor) were used to filter the nanoparticles during the purification step. HNO_3 (Carl Roth GmbH, Rotipuran Suprapur 69 wt%), H_2O_2 (30%), HF (Sigma-Aldrich, Suprapur 40 wt%) H_3BO_3 (Merck, 5 wt%) were used for the sample preparation for ICP-OES measurements.

Methods

Synthesis of ZrO_2 core nanocrystals

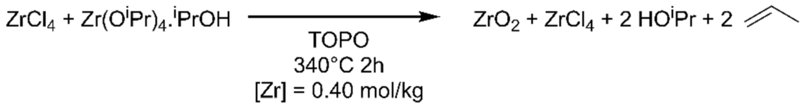

Zirconia nanocrystals are synthesized and purified following the procedure previously reported (with one difference: zirconium chloride was used instead of its THF complex).[5] Briefly, $ZrCl_4$ (2 mmol, 0.466 g), $Zr(O^iPr)_4$ ^iPrOH (2 mmol, 0.775 g) and recrystallized TOPO (10 g) are mixed at room temperature and heated up under argon to 340°C for 2 hours. After the reaction is completed, the nanocrystals are purified using acetone as non solvent and toluene as solvent as previously described.[5] Finally, the particles are dispersed in 10 mL cyclohexane and filtered with a PTFE membrane. Yield: 285.9 mg, 96%, calculated assuming that maximum 2 mmol of ZrO_2 can form since the 2 mmol $ZrCl_4$ is retrieved again after reaction, and considering that 17% of the mass is assigned to organic ligand (as determined by TGA).

Synthesis of ZrO_2:Eu core nanocrystals

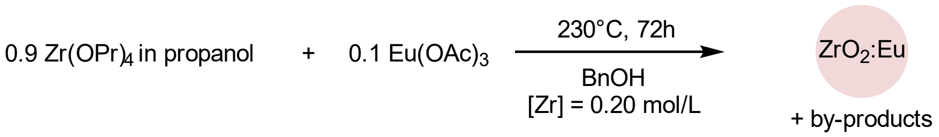

The assembly of a 125 mL autoclave was carried out in a glovebox (O_2 < 3 ppm, H_2O < 1 ppm). To the Teflon liner, zirconium(IV) propoxide in propanol (9 mmol, 4 mL) was added to benzyl alcohol (41.4 mL) and $Eu(OAc)_3$ (1 mmol, 0.343 g), the autoclave was closed and transferred to a muffle furnace. The temperature was increased at a rate of 3.8°C/min and kept at 230°C for 72h. After the synthesis, the powder is washed with 30 mL ether. The nanocrystals are de-aggregated by functionalizing the surface with 750 mg dodecanoic acid, by sonicating for 2h 25min in 5 mL toluene, precipitating three times once with 10 mL, then twice with 15 mL acetone as antisolvent, and collected by centrifugation. Once purified, the nanocrystals are dissolved in cyclohexane for storage. These particles, after being dried, are used as seeds. Yield: 1.114 g, 60.8%, calculated considering 29% of organic fraction determined by TGA.

Synthesis of ZrO_2/HfO_2 core/shell nanocrystals

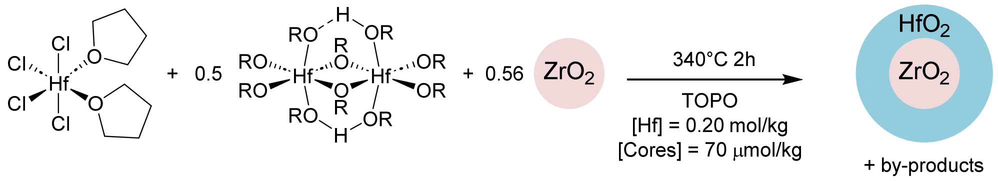

To shell the zirconia nanocrystals the procedure reported by De Keukeleere et al.[5] has been modified. In a nitrogen filled glovebox, a 25 mL three-neck flask is loaded with a stirring bard and reagents in the exact following order $Hf(O^iPr)_4$ ^iPrOH (0.5 mmol, 0.238 g), recrystallized TOPO (2.5 g), $HfCl_4(THF)_2$ (0.5 mmol, 0.233 g), recrystallized TOPO (2.5 g) and the 4 nm dried ZrO_2 nanoparticles (41.7 mg seed particles (including ligands), 0.28 mmol ZrO_2). The total amount of TOPO is 5 g. The flask is closed on one end with a septum, in the middle with a condenser, and the third neck with a thermowell. After being connected to the Schlenck line and filled with Argon, the temperature is raised slowly to 340°C (it takes around 15-20 minutes to reach it) and held at this temperature. After 2 hours the reaction is completed, the reaction mixture is cooled to 100°C and toluene (2 mL) is injected. The nanoparticles are purified with acetone (1:2 in volume) and toluene/cyclohexane (2 mL) following the previously reported procedure.[5] To store the nanoparticles, they are suspended in 5 mL cyclohexane. Yield: 62.8 mg, 14% of which is the organic fraction determined by TGA.

Note that the hafnium isopropoxide precursor was synthesized following the procedure reported by Bradley *et al.*[2] and Dhaene *et al.*[3] When we used hafnium isopropoxide purchased from Strem Chemicals, we observed secondary nucleation of HfO_2 (Figure S15 and S16).

Synthesis of ZrO_2:Eu/ZrO_2 core/shell nanocrystals

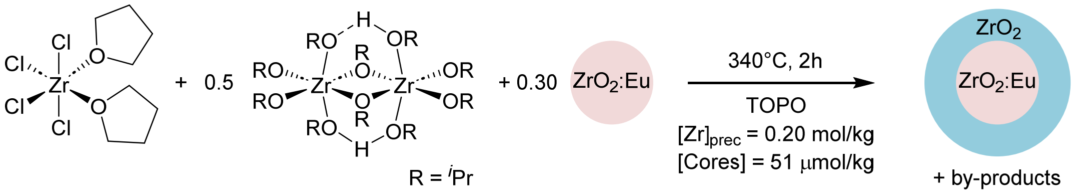

Same procedure as the one previously described for the HfO_2 shelling of ZrO_2 but the reaction was 10-fold scaled up. ZrO_2:Eu seeds capped with dodecanoic acid (276 mg seed particles (including ligands), 1.52 mmol ZrO_2:Eu) were added to $ZrCl_4(THF)_2$ (5 mmol, 1.886 g), $Zr(O^iPr)_4$ ^iPrOH (5 mmol, 1.938 g) and recrystallized TOPO (50 g). Yield 1.026 g, 16% of which is the organic fraction determined by TGA.

Sample preparation and measurement with ICP-OES

Dry powders of the respective particles were prepared in duplicates, weighed in amounts of ≈1-2 mg and digested using a two-step procedure, suitable for total dissolution of both ZrO_2 and rare earth element dopants.[6] In brief, for the first step, 2 ml HNO_3 (69 wt%), 1 ml H_2O_2 (30%) and 0.3 ml HF (40 wt%) were added to the sample in a PTFE digestion tube. The mixture was then digested in a pressurized microwave (UltraClave, MLS GmbH) at 240 °C and 100 bar for 10 min. For the second step, 2.4 ml H_3BO_3 (5 wt%) was added to the samples and the digestion in the microwave carried out a second time. Afterwards, the samples were transferred to 50 ml falcon tubes and filled to the mark with ultra-pure water. The amount of Zr and Eu in these samples was then determined using inductively-coupled plasma optical emission spectroscopy (ICP-OES). The elements were determined using external calibration with certified standards (Inorganic ventures) at wavelengths of 343.823 nm (Zr) and 420.504 nm (Eu).

Sample preparation for TEM

The nanoparticles were dissolved in cyclohexane to obtain solutions with concentrations of 1 mg/mL. Two drops of the solution were deposited on a carbon grid Quantifoil R 1.2/1.3 Cu 200 + 2 nm C.

Instrumentation

Transmission Electron Microscopy (TEM) HR-TEM imaging was carried out in JEOL JEM-F200 operated in the TEM-mode at a beam energy of 200kV. HAADF-STEM images were acquired on an aberration-corrected ThermoFisher Spectra 30-300 S/TEM operated at 300 kV. Samples were prepared by drop-casting diluted solutions of NCs onto ultrathin carbon film-coated 400 mesh lacey carbon copper grids. Atomic resolution images were acquired on a high-angle annular dark field (HAADF) detector with a current of 30 pA and a beam convergence semiangle of 25 mrad. The EDX signal was collected by a Dual-X system comprising two detectors, one on either side of the sample, for a total acquisition solid angle of 1.76 sr.

Photoluminescence Spectroscopy (PL) spectra in liquid state were acquired at room temperature using the Spectrofluorometer SF5 (Edinburgh Instruments), with a Standard Cuvette holder SC-05 using quartz cuvettes and a detection angle of 90 degrees. The nanocrystals were dispersed in cyclohexane with absorbance of 0.1 at the excitation wavelength. The samples did not show any sign of turbidity or scattering. A xenon lamp was used as excitation source. Data were processed with the software Fluoracle. Spectra in solid state (powders) were recorded at 10K or at room temperature with an FS920 spectrofluorometer (Edinburgh Instruments), in combination with an Optistat CF cryostat (Oxford Instruments).

Dynamic Light Scattering (DLS) data were acquired on a Malvern Zetasizer ZS instrument in backscattering mode at 25°C. Measurements were performed in glass cuvettes and recorded three times after equilibrating for 120 seconds. Data processing was performed using the software Malvern ZS Explorer by using the "general purpose" analysis model.

Powder X-ray Diffraction (XRD) was recorded using a SmartLab diffractometer system 3.1.03. (Rigaku, Tokyo, Japan) equipped with a HyPix-3000 detector and a knife edge. Samples were placed on a low-background silica sample holder. Measurements were conducted in the range of 3°-90° 2θ at 27°/min with steps of 0.01°.

Nuclear Magnetic Resonance (NMR) measurements were recorded at 298K on Bruker UltraShield 500 spectrometer operating at a frequency of 500.13 MHz. ^{31}P NMR spectra were acquired using inverse gated decoupling and 4000 scans. ^{31}P NMR spectra were processed with a line broadening of 50 Hz. All resonances are background-corrected. Chemical shifts (δ) are given in parts per million (ppm), and the residual solvent peak was used as an internal standard (C_6D_6: δH = 7.16 ppm).

Thermogravimetric Analysis (TGA) was performed under air on a TGA5500 (TA instruments) instrument. The samples were heated to 800 °C at a ramping rate of 5 °C/min. At the end an isotherm of 15 min is given to ensure that all the organics are burned out.

Inductively-Coupled Plasma Optical Emission Spectroscopy (ICP-OES) measurements were performed on a 5110 ICP-OES (Agilent Switzerland AG, Basel, Switzerland) apparatus.

Structural characterization with X-ray total scattering and Pair Distribution Function (PDF) analysis. X-ray total scattering data were collected at beamline P21.1 at PETRA III/DESY in Hamburg, Germany, and at beamline 11-ID-B at the Advanced Photon Source, Argonne National Laboratory, USA. Measurements were carried out at room temperature on samples prepared in 1 mm polyamide kapton tubes in rapid acquisition mode using a large-area 2D PerkinElmer detector (2048 x 2048 pixels, 200 µm x 200 µm pixel size) with a sample-to-detector distance of 350 mm (P21.1) and 180 mm (11-ID-B). The incident wavelength of the X-rays was λ = 0.1222 Å (P21.1) and λ = 0.2116 Å (11-ID-B) and the measurement exposure time was 180s (P21.1) and 300s (11-ID-B). To calibrate the experimental setup a CeO_2 (P21.1) respectively a Si (11-ID-B) standard was used, while the scattering pattern of the empty kapton was used as background. The data were integrated using pyFAI[7] and the PDF spectra were obtained using xPDFSuite[8] with PDFgetX3 with Q_{max} = 21.0 (P21.1), Q_{max} = 22.0 (11-ID-B), Q_{min} = 0.8 and R_{poly} = 0.90. To reduce the data, the chemical composition of the nanoparticles was used.

Figures

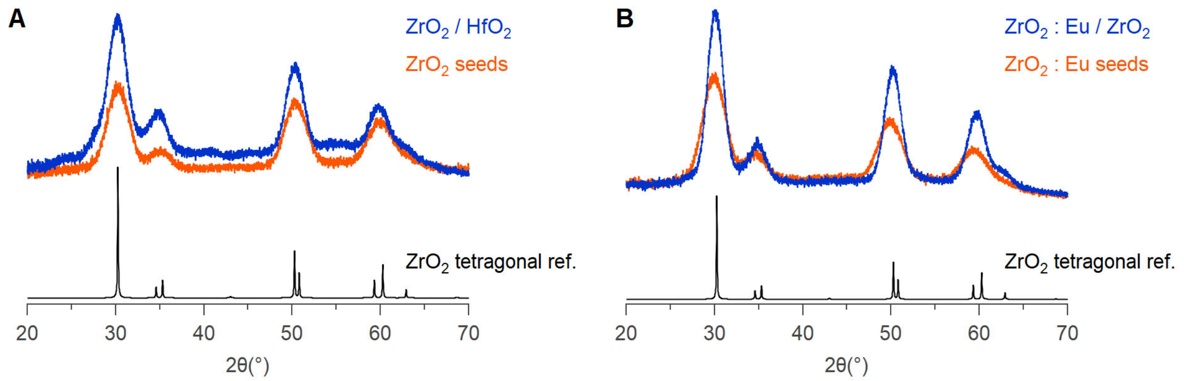

Figure S1: Powder XRD spectra of (A) core ZrO_2 and core/shell ZrO_2/HfO_2 nanocrystals (B) ZrO_2:Eu and core/shell ZrO_2:Eu/ZrO_2, together with the ZrO_2 tetragonal reference.[9]

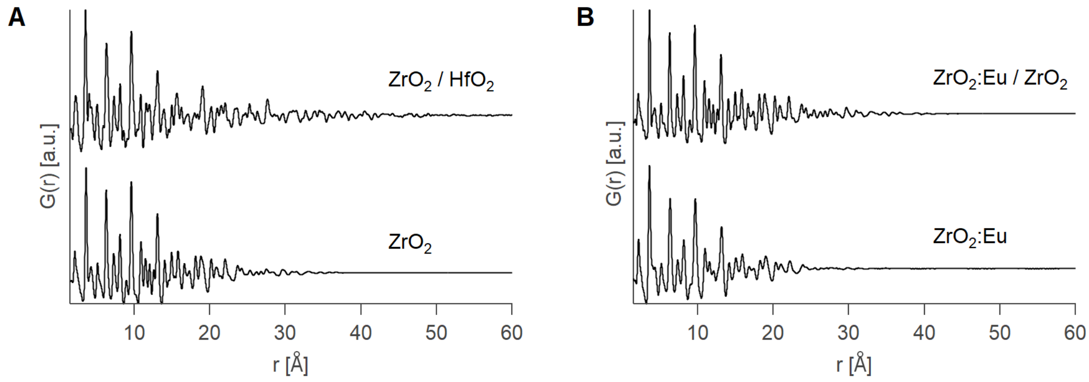

Figure S2: Pair Distribution Function modelfree analysis of (A) core ZrO_2 and core/shell ZrO_2/HfO_2 nanocrystals (B) ZrO_2:Eu and core/shell ZrO_2:Eu/ZrO_2.

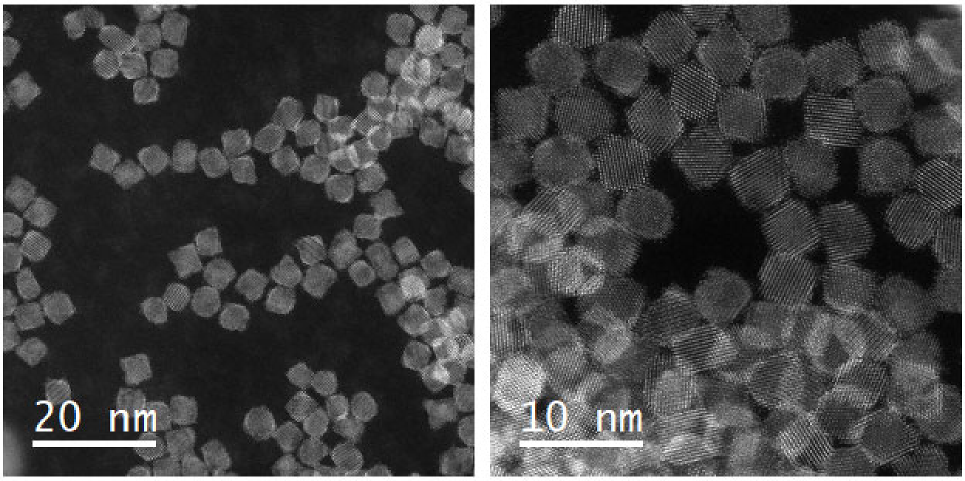

Figure S3: HAADF STEM image showing the morphology of the core/shell ZrO_2/HfO_2 nanoparticles. The NPs have a faceted shape. From the image contrast, the presence of a core/shell structure can be recognized: the shell appears brighter than the core due to the different atomic numbers of zirconium and hafnium. Three plasma cleaning cycles of 4 seconds each were performed to minimize the contamination due to the beam exposure.

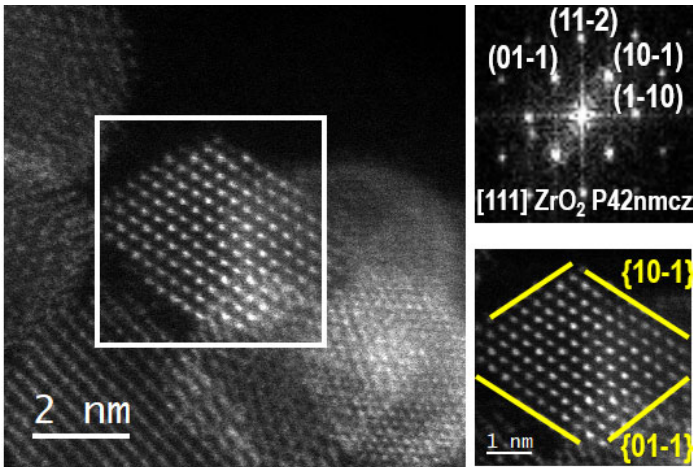

Figure S4: Atomic resolution HAADF STEM images showing the structure of the ZrO_2/HfO_2 core/shell nanoparticles. Power spectrum analysis shows the faceted shape of the NPs, exposing mainly {01-1} and {10-1} surfaces.

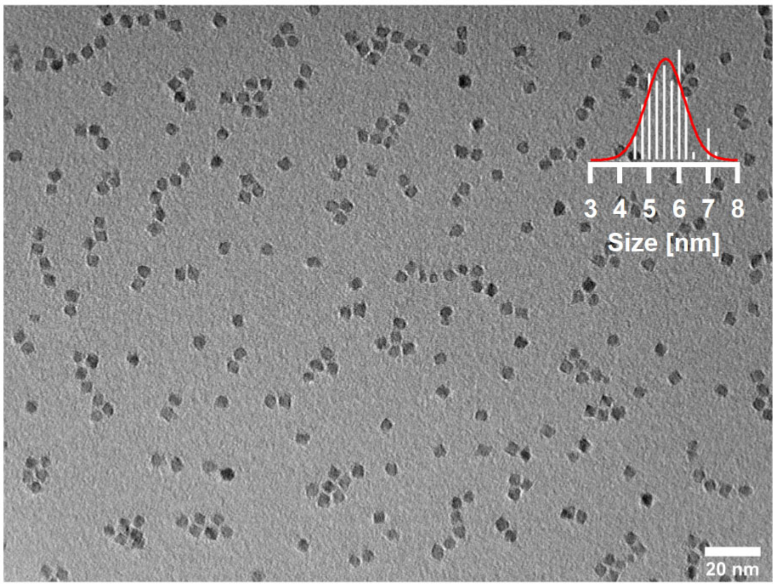

Figure S5: BF TEM Image of ZrO_2/ZrO_2 core/shell nanoparticles synthesized using $Zr(O^iPr)_4$ ^iPrOH (0.5 mmol, 0.194 g), $ZrCl_4(THF)_2$ (0.5 mmol, 0.189 g), recrystallized TOPO (5 g) and 3.8 nm dried ZrO_2 nanoparticles (41.4 mg seed particles (including ligands), 0.28 mmol ZrO_2). After counting 80 nanoparticles, the average diameter is 5.5 nm. The final size reflects a three-fold volume increase compared to the initial seeds (d = 3.8 nm), consistent with the stoichiometry of the reaction.

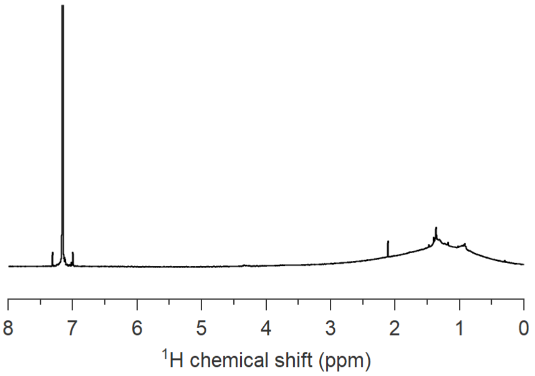

Figure S6: ^{1}H NMR spectrum of dodecanoic acid capped ZrO_2:Eu core nanoparticles in C_6D_6.

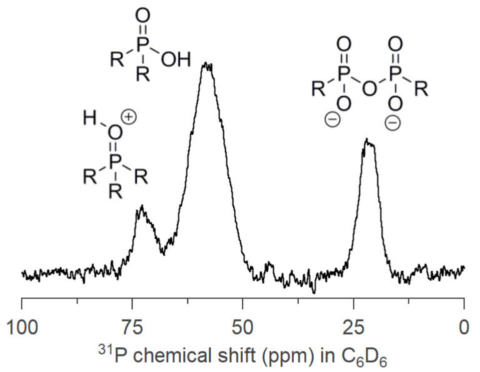

Figure S7: ^{31}P NMR spectrum of ZrO_2:Eu/ZrO_2 core/shell nanocrystals in C_6D_6.

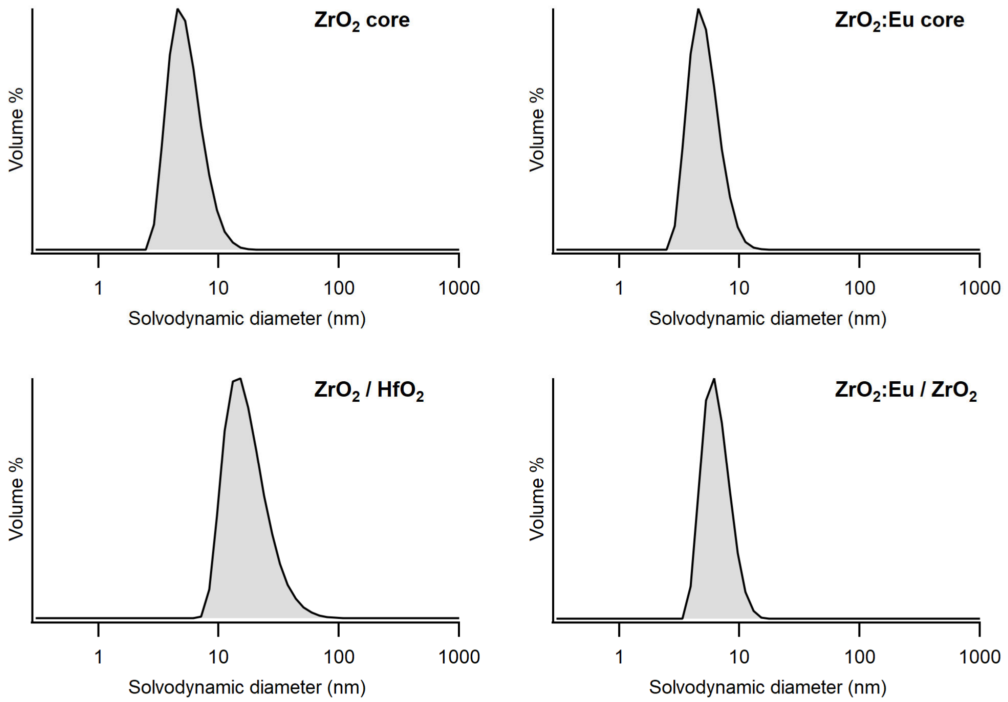

Figure S8: DLS volume distribution of the core and core/shell nanocrystals in cyclohexane.

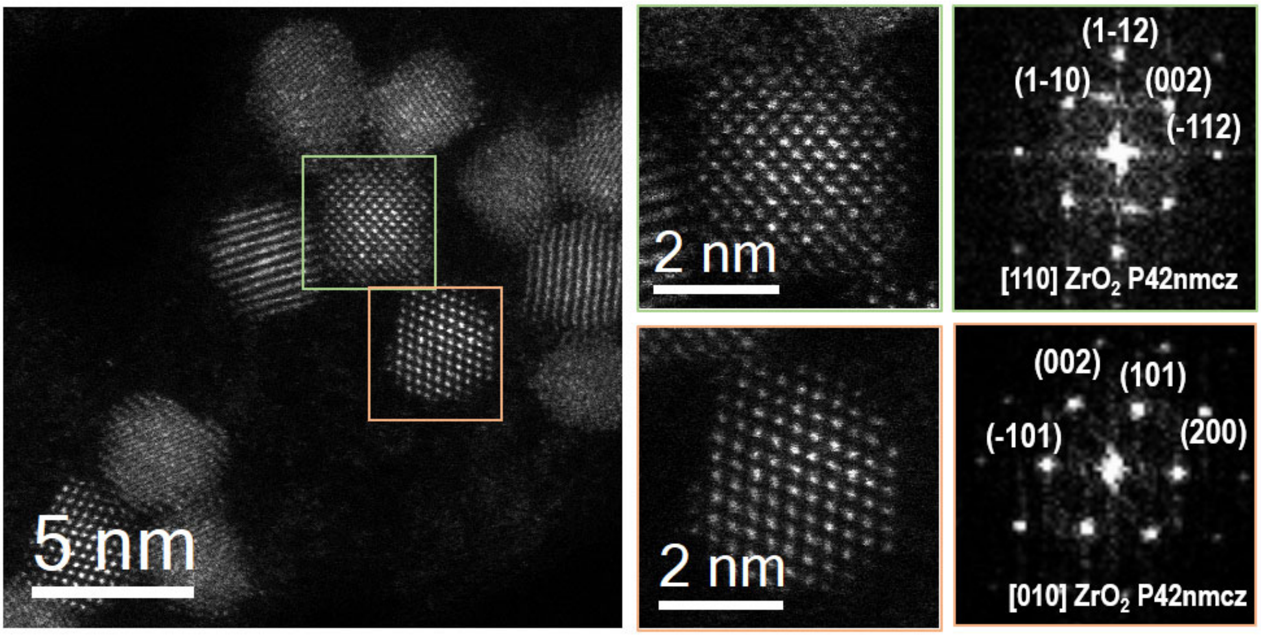

Figure S9: Atomic resolution HAADF STEM images showing the structure of the ZrO_2:Eu core nanoparticles. Power spectrum analysis shows that the nanoparticles possesses the P 42/nmc Z (137) space group. The nanoparticles are imaged along the [110] and the [010] zone axes. The low europium concentration does not affect the crystalline structure of the nanoparticles.

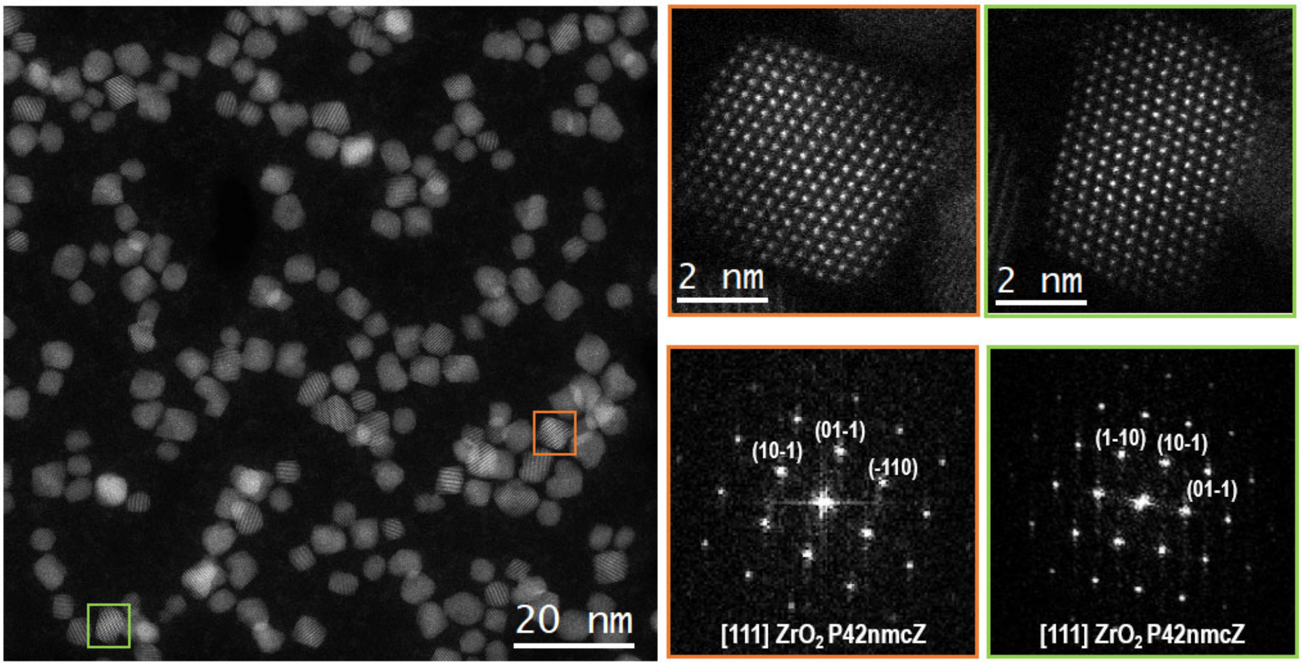

Figure S10: Atomic resolution HAADF STEM images showing the structure of the ZrO_2:Eu/ZrO_2 core/shell nanoparticles. Power spectrum analysis shows that the nanoparticles possesses tetragonal crystal structure with the P 42/nmc Z (137) space group. The highlighted nanoparticles are imaged along the [111] zone axes.

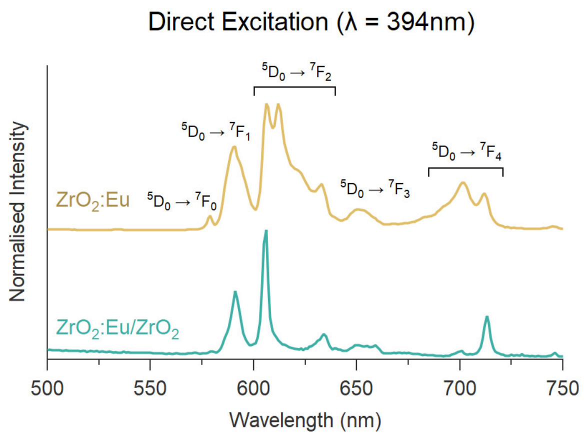

Figure S11: Photoluminescence emission spectra of ZrO_2:Eu cores and ZrO_2:Eu/ZrO_2 core/shells, measured under direct excitation at 394 nm at room temperature in cyclohexane.

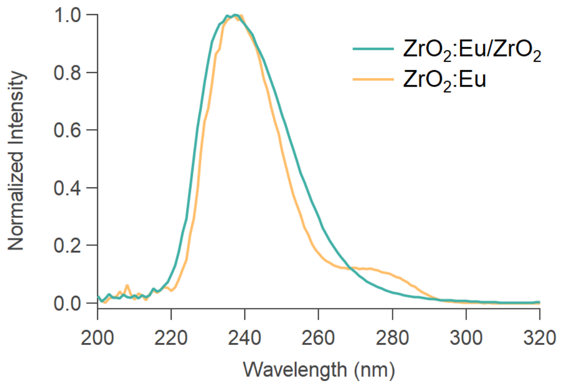

Figure S12: Photoluminescence excitation spectra of ZrO_2:Eu cores and ZrO_2:Eu/ZrO_2 core/shells, measured at 606 nm at room temperature in cyclohexane.

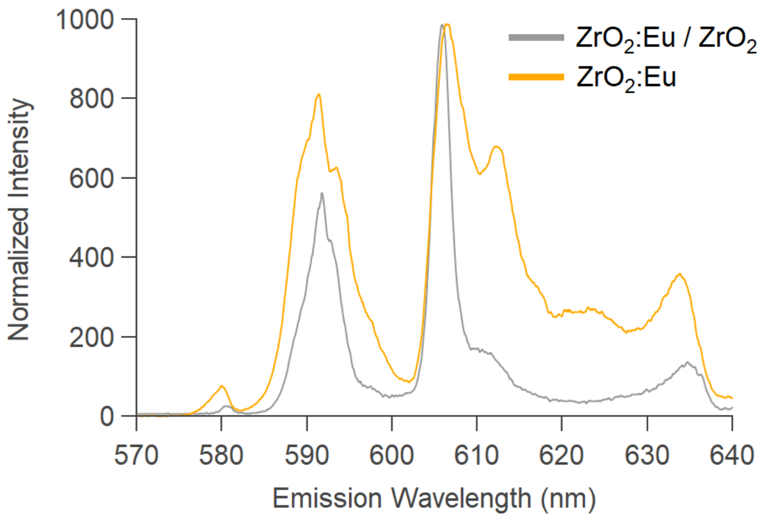

Figure S13: Solid state emission spectra of core ZrO_2:Eu (orange) and core/shell ZrO_2:Eu/ZrO_2 (grey) nanocrystals upon excitation at 260/280 nm measured at 10K. The difference in position of the $^5D_0$-$^7F_0$ transition around 580 nm is due to the different environments present in the core while it is narrower, closer to a single site in the case of the shelled nanoparticles.

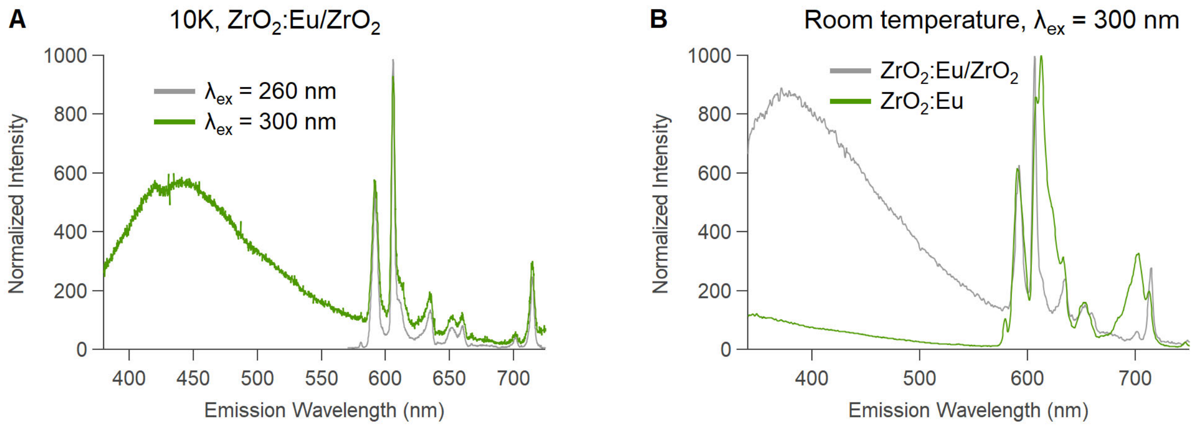

Figure S14: (A) Solid state emission spectra of ZrO_2:Eu/ZrO_2 nanocrystals upon excitation at 260 nm (grey) and 300 nm (green) measured at 10K. A strong broadband contribution is detected when the sample is excited at 300 nm, while by exciting at 260 nm the spectrum shows predominantly the Eu^{3+} contribution. (B) Solid state emission spectra of ZrO_2:Eu and ZrO_2:Eu/ZrO_2, upon excitation at 300 nm and measured at room temperature. The broadband emission is clearly detected in ZrO_2:Eu/ZrO_2, while hardly present in ZrO_2:Eu. Notice that at the used excitation wavelength of 300nm, the Eu^{3+} centers are not efficiently excited, in contrast to the defect emission. The broadband emission in the ZrO_2:Eu/ZrO_2 is also visually apparent by observation of white emission under a UV lamp, while the ZrO_2:Eu seeds glow only with the red emission from europium.

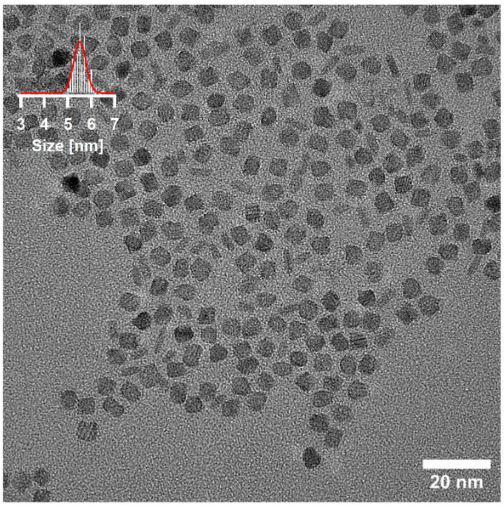

Figure S15: BF TEM Image of ZrO_2/HfO_2 core/shell nanoparticles synthesized using $Hf(O^iPr)_4$ ^iPrOH (0.44 mmol, 0.207 g) purchased by Strem Chemicals, $HfCl_4(THF)_2$ (0.5 mmol, 0.233 g), recrystallized TOPO (5 g) and 4 nm dried ZrO_2 nanoparticles (0.27 mmol ZrO_2, 40 mg (including ligands)). After counting 130 nanoparticles, the average diameter size is 5.5 ± 0.3 nm (μ ± σ).

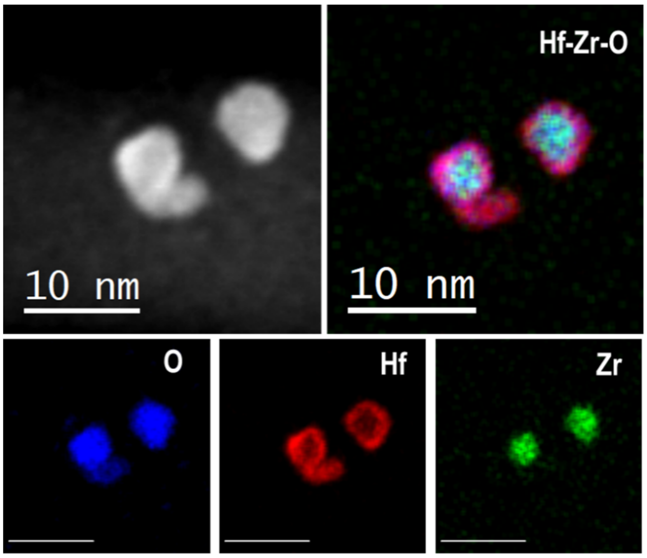

Figure S16: HAADF STEM and corresponding EDX compositional maps of the particles represented in Figure S15, showing the nanorods being pure HfO_2.

References

(1) Manzer, L.; Deaton, J.; Sharp, P.; Schrock, R. 31. Tetrahydrofuran Complexes of Selected Early Transition Metals. *Inorganic Syntheses* **1982**, *21*, 135-140.

(2) Bradley, D.; Mehrotra, R.; Wardlaw, W. 330. Hafnium alkoxides. *Journal of the Chemical Society (Resumed)* **1953**, 1634-1636.

(3) Dhaene, E.; Seno, C.; De Roo, J. Synthesis of zirconium(IV) and hafnium(IV) isopropoxide, *sec*-butoxide and *tert*-butoxide. *Dalton Transactions* **2024**, doi.org/10.1039/D4DT01280A.

(4) Owen, J. S.; Park, J.; Trudeau, P.-E.; Alivisatos, A. P. Reaction chemistry and ligand exchange at cadmium− selenide nanocrystal surfaces. *Journal of the American Chemical Society* **2008**, *130* (37), 12279-12281.

(5) De Keukeleere, K.; Coucke, S.; De Canck, E.; Van Der Voort, P.; Delpech, F.; Coppel, Y.; Hens, Z.; Van Driessche, I.; Owen, J. S.; De Roo, J. Stabilization of Colloidal Ti, Zr, and Hf Oxide Nanocrystals by Protonated Tri-n-octylphosphine Oxide (TOPO) and Its Decomposition Products. *Chemistry of Materials* **2017**, *29* (23), 10233-10242.

(6) Kaegi, R.; Gogos, A.; Voegelin, A.; Hug, S. J.; Winkel, L. H.; Buser, A. M.; Berg, M. Quantification of individual Rare Earth Elements from industrial sources in sewage sludge. *Water research X* **2021**, *11*, 100092.

(7) Ashiotis, G.; Deschildre, A.; Nawaz, Z.; Wright, J. P.; Karkoulis, D.; Picca, F. E.; Kieffer, J. The fast azimuthal integration Python library: pyFAI. *Journal of applied crystallography* **2015**, *48* (2), 510-519.

(8) Yang, X.; Juhas, P.; Farrow, C. L.; Billinge, S. J. xPDFsuite: an end-to-end software solution for high throughput pair distribution function transformation, visualization and analysis. *arXiv preprint arXiv:1402.3163* **2014**.

(9) Howard, C. J.; Kisi, E. H.; Roberts, R. B.; Hill, R. J. Neutron diffraction studies of phase transformations between tetragonal and orthorhombic zirconia in magnesia-partially-stabilized zirconia. *Journal of the American Ceramic Society* **1990**, *73* (10), 2828-2833.
